# Structure–Function Aspects of PstS in Multi-Drug–Resistant *Pseudomonas aeruginosa*

**DOI:** 10.1371/journal.ppat.0040043

**Published:** 2008-02-15

**Authors:** Olga Zaborina, Christopher Holbrook, Yimei Chen, Jason Long, Alexander Zaborin, Irina Morozova, Hoylan Fernandez, Yingmin Wang, Jerrold R Turner, John C Alverdy

**Affiliations:** 1 Department of Surgery, Pritzker School of Medicine, University of Chicago, Chicago, Illinois, United States of America; 2 Department of Biochemistry and Molecular Biology, University of Chicago, Chicago, Illinois, United States of America; 3 Department of Pathology, University of Chicago, Chicago, Illinois, United States of America; Harvard Medical School, United States of America

## Abstract

The increasing prevalence of multi-drug–resistant (MDR) strains of Pseudomonas aeruginosa among critically ill humans is of significant concern. In the current study, we show that MDR clinical isolates of P. aeruginosa representing three distinct genotypes that display high virulence against intestinal epithelial cells, form novel appendage-like structures on their cell surfaces. These appendages contain PstS, an extracellular phosphate binding protein. Using anti-PstS antibodies, we determined that the PstS-rich appendages in MDR strains are involved in adherence to and disruption of the integrity of cultured intestinal epithelial cell monolayers. The outer surface–expressed PstS protein was also identified to be present in P. aeruginosa MPAO1, although to a lesser degree, and its role in conferring an adhesive and barrier disruptive phenotype against intestinal epithelial cells was confirmed using an isogenic ΔPstS mutant. Formation of the PstS rich appendages was induced during phosphate limitation and completely suppressed in phosphate-rich media. Injection of MDR strains directly into the intestinal tract of surgically injured mice, a known model of phosphate limitation, caused high mortality rates (60%–100%). Repletion of intestinal phosphate in this model completely prevented mortality. Finally, significantly less outer surface PstS was observed in the MPAO1 mutant ΔHxcR thus establishing a role for the alternative type II secretion system Hxc in outer surface PstS expression. Gene expression analysis performed by RT-PCR confirmed this finding and further demonstrated abundant expression of *pstS* analogous to *pa5369*, *pstS* analogous to *pa0688/pa14–55410*, and *hxcX* in MDR strains. Taken together, these studies provide evidence that outer surface PstS expression confers a highly virulent phenotype of MDR isolates against the intestinal epithelium that alters their adhesive and barrier disrupting properties against the intestinal epithelium.

## Author Summary

The resistance of bacteria to multiple antibiotics is a major problem in critically ill patients who often become colonized by highly lethal pathogens such as *Pseudomonas aeruginosa.* During the course of critical illness, as many as 50% of patients' intestinal tracts become colonized with *P. aeruginosa,* with as many as 30% of strains being resistant to multiple antibiotics. Concomitantly, critical illness is characterized by acute depletion of phosphate, which itself has been shown to be an independent predictor of infection-related mortality. In the present study we determined that during low phosphate conditions, highly virulent multi-antibiotic–resistant strains of P. aeruginosa isolated from critically ill patients produce an abundance of the phosphate-binding protein, PstS, located on extracellular finger-like structures. These PstS rich appendages participate in the binding of P. aeruginosa to intestinal lining cells and may allow P. aeruginosa to acquire phosphate from its host while remaining at arm's length from the host immune system. This clever tactic may be one example by which successful opportunistic pathogens such as P. aeruginosa survive within complex ecological niches such as the intestinal tract and harm their hosts during the course of critical illness.

## Introduction

Infection due to P. aeruginosa continues to be a major cause of mortality among critically ill and immuno-compromised patients despite the development of newer and more powerful antibiotics. Both the immunoevasive nature of P. aeruginosa as well as its acquisition of multi-drug resistance makes elimination of this organism a particular challenge. Multi-drug–resistant (MDR) strains of P. aeruginosa, defined as resistant to at least three of the following antibiotics: ceftazidime, imipenem, gentamicin or ciprofloxacin, are often isolated from patients exposed to prolonged intensive care-type therapies [1]. Yet antibiotic resistance itself does not confer enhanced virulence [2], and therefore the ability to discriminate between virulent versus non-virulent phenotypes among colonizing multi-drug resistant isolates would be a major step in predicting the particular threat of a colonizing strain of *P. aeruginosa.* The primary site of colonization and a frequent source of subsequent infection of P. aeruginosa is the gastrointestinal tract reservoir, where as many as 50% of critically ill patients are colonized within 3 days of admission with as many as 30% of strains displaying antibiotic resistance [3]. Yet little is known about the behavior of these pathogens in this site, especially those that are multi-drug resistant. We recently screened several strains of MDR isolates from hospitalized patients and characterized their virulence against the intestinal epithelium using an *in vitro* model of cultured intestinal epithelial monolayers [2]. The majority of strains (60%) were found to be either attenuated or have no effect in their ability to adhere to or disrupt the integrity of the intestinal epithelium. However several strains representing three distinct genotypes, showed extremely high adherence capacity and a profound ability to disrupt the barrier function of cultured intestinal epithelial cells. These strains harbored the *exoU* gene, known to encode the most toxic effector protein of the type III secretion apparatus, thus possibly explaining their extreme toxicity against cultured intestinal epithelial cells. However, *exoU* expression is dependent on contact to host epithelial cells, and as recently shown with the *exoU* positive strain P. aeruginosa PA103 [4], lack of adherence leads to a loss of cytotoxicity against cultured epithelial cells [5] despite an intact *exoU* gene. Therefore we studied selected *exoU* positive MDR strains of P. aeruginosa displaying unusually high adherence and disrupting properties against the intestinal epithelium and determined whether surface structures might exist to explain their enhanced adhesiveness to cultured intestinal epithelial cells. In this report we show that these strains express previously un-described appendages that contain significant quantities of PstS, a high affinity phosphate binding protein. We characterized the structural and functional aspects of these PstS rich appendages and determined that they play a significant role in the adherence to and disruption of intestinal epithelial cells. Outer surface expression of PstS rich appendages was induced under low phosphate conditions and suppressed in high phosphate media. Lethality assays in a mouse model of gut-derived sepsis in which low phosphate conditions are known to exist, demonstrated high lethality rates that were completely abrogated when mice were supplemented with intestinal phosphate. Taken together, these data provide evidence that low phosphate conditions increase the presence of PstS rich appendages on MDR P. aeruginosa whose presence facilitates binding to the intestinal epithelium and whose expression *in vivo* may play a significant role in the development of gut-derived sepsis in critically ill patients.

## Results

### Highly Virulent MDR P. aeruginosa Express Novel Appendage-Like Structures

**Figure 1:**
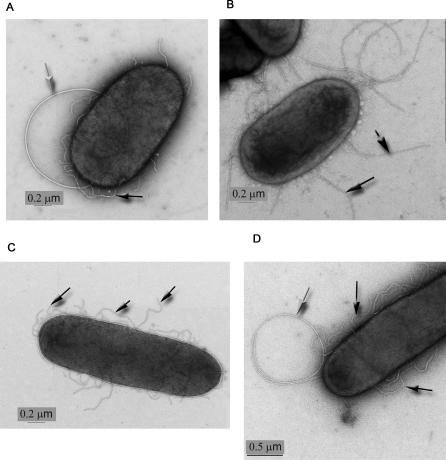
Multi-Drug–Resistant P. aeruginosa Clinical Isolates Display Unusual Appendage-Like Structures on the Cell Surface Transmission electron microscopy (TEM) images of MDR virulent clinical isolates at magnification of 15,000 × 1.4. (A) Strain MDR1. (B) Strain MDR13. (C) Strain MDR25. (D) Strain MDR26. Novel appendage-like structures are shown by black arrows. For comparison, flagella are indicated by grey arrows.

In our previous work we screened consecutive MDR P. aeruginosa clinical isolates and identified a subset of strains that displayed a highly destructive phenotype against cultured intestinal epithelial cells (Caco-2_ bbe_) [2]. Among this subset of isolates, high swimming motility, increased adhesiveness to Caco-2 monolayers, and the presence of the *exoU* gene predicted a cytotoxic phenotype against the intestinal epithelium [2]. In the present study, we screened these highly adhesive MDR clinical isolates by their cell surface morphology using electron microscopy, and identified appendage-like structures on the surfaces of the most cytotoxic isolates (1, 13, and those of genotype 20) (Figure 1A–1D and Table 1). The identified appendages were 20 nm in diameter, up to 500 nm in length, and were visually distinct from flagella (Figure 1A and 1D). The identified appendages were not detected on any of the remaining clinical isolates of the previously reported series of strains (see Figures S1 and S2).

**Table 1:**
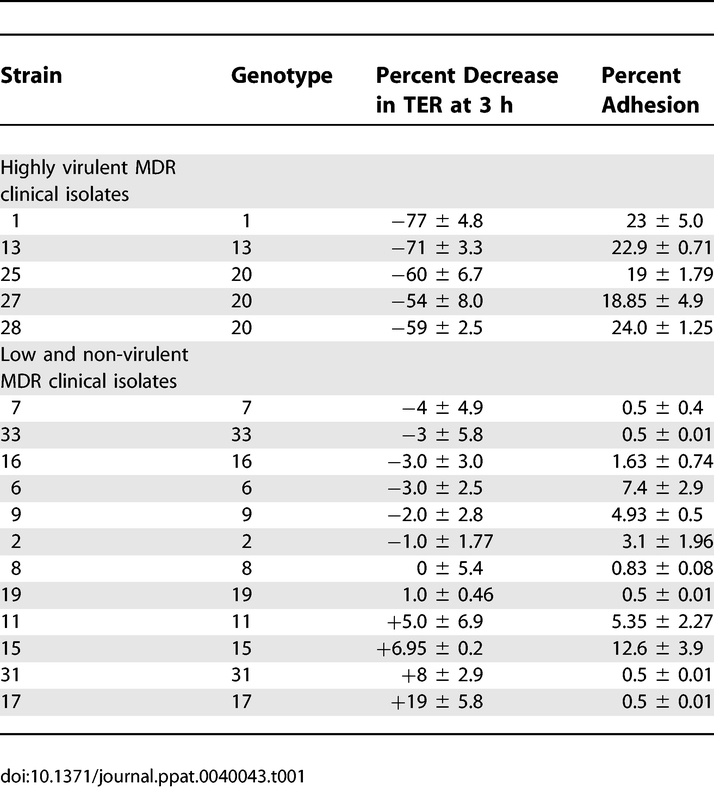
Virulence Characteristics of MDR P. aeruginosa Clinical Isolates Assessed by Their Ability to Adhere to (% Adhesion) and Alter the Transepithelial Electrical Resistance (% Decrease in TER) of Caco-2 Cells [2]

### Identification of Proteins Involved in the Formation of Novel Appendages

**Figure 2:**
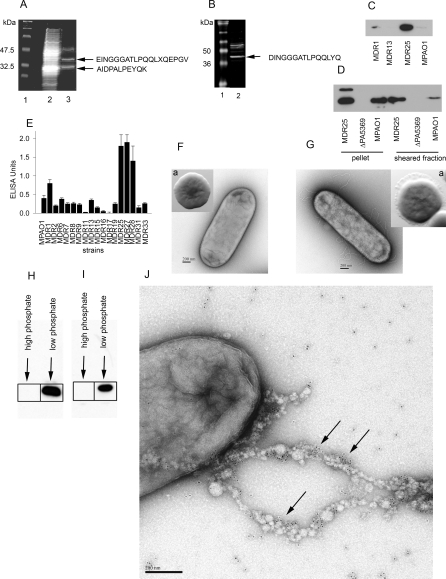
Identification of Appendage-Like Structures (A, B) Fractionation of sheared cell surface proteins by 10% Tris-Glycine SDS-PAGE and identification of abundant proteins by N-terminal sequencing. (A), Lane 1, molecular weight standards (Bio-Rad, broad range, pre-stained); lane 2, strain MDR25; lane 3, strain MDR1. Black arrows show proteins of strain MDR1 subjected to N-terminal sequencing. (B) Lane 1, molecular weight standards (Invitrogen, SeeBlue Plus 2 pre-stained); lane 2, strain 13. Black arrow shows protein subjected to N-terminal sequencing. (C) Immunoblot analysis of cell surface sheared proteins isolated from strains MDR1, MDR13, MDR25, and MPAO1 by using affinity purified polyclonal anti-PA5369 antibody. (D) Immunoblot analysis of pellet and sheared surface proteins from strains MPAO1, MPAO1 derivative PA5369 mutant, and clinical strain MDR25. Colonies were collected and suspended in 200 μl PBS up to final density of 5.0 (600 nm) followed by vortexing for 2 min and centrifuging at 5,000*g*, 5 min. Sheared material in 10 μl of supernatant was denatured by boiling with 10 μl sample buffer and 10 μl was loaded on gel wells to be separated by 10% glycine SDS-PAGE. The remaining pellet was resuspended in 50 μl of sample buffer, boiled, and 2 μl were loaded on gel wells. PstS protein was identified by immunoblot using anti-PA5369 antibody. (E) Enzyme-linked immunosorbent assay (ELISA) to detect PA5369-like protein on cell surfaces of MPAO1 and MDR clinical P. aeruginosa strains. Data are mean ± SD (*n* = 3). (F, G) TEM images of strain MDR25 grown on (F) PIA supplemented with 1 mM K-phosphate buffer and (G) on PIA. (H, I) Immunoblot of (H) sheared surface proteins and (I) cell pellet proteins in strain MDR25 grown on PIA (low phosphate) versus PIA supplemented with 1 mM K-phosphate buffer (high phosphate). (J) Immuno-electron microscopy of strain MDR25. Gold spots identified with arrows indicate the protein recognized by anti-PA5369 antibody on cell surface structures.

To identify proteins involved in the formation of the visualized appendages, surface-associated proteins were obtained by extensive vortexing of bacterial cells grown on *Pseudomonas* isolation agar (PIA), denatured by boiling with sample buffer and then separated by 10% Tris-glycine SDS-PAGE. Figures 2A and 2B show the presence of abundant protein bands at an approximate MW of 32 kDa from surface sheared proteins in strain MDR25 (Figure 2A, lane 2); 32 and 40 kDa bands from strain MDR1 (Figure 2A, lane 3); and a 40 kDa band from strain MDR13 (Figure 2B, lane 2). Proteins were transferred to a PVDF membrane and N-terminal sequencing of the 32 and 40 kDa proteins in strain MDR1 and the 40 kDa protein in strain MDR13 were performed with ABI-Procise cLC Protein Sequencer (Mayo Proteomics Research Center). The N-terminal peptide sequence of the 32 and 40 kDa proteins in strain MDR1 were found to be AIDPALPEYQK and EINGGGATLPQQLXQEPGV, respectively. The N-terminal peptide sequence of 40 kDa protein in strain MDR13 was identified as DINGGGATLPQQLYQ. The peptide sequences were searched with BLAST, and the best hit of the 32 kDa band sequence was found to be the PstS protein PA5369 in P. aeruginosa PAO1. The sequence AIDPALPEYQK was located to aa 25–35 on ORF PA5369. PA5369 was demonstrated to contain a cleavable type I signal peptide of 24 aa [6], therefore the 32 kDa protein in strain MDR1 might correspond to periplasmic orthologous protein PA5369 in PAO1. The best hit of the 40 kDa band sequence in both strains 1 and 13 was found to be PA55410 from P. aeruginosa PA14 (http://v2.pseudomonas.com/getAnnotation.do?locusID=PA14_55410). The sequence DINGGGATLPQQLYQ was located to aa 24–38 on ORF PA55410. Orthologous to PA55410, PA0688 protein in PAO1 was also demonstrated to contain a cleavable type I signal peptide of 23 aa, MFKRSLIAASLSVAALVSAQAMA [6], which was 100% identical to N-terminus of PA55410. Therefore the 40 kDa proteins in strains 1 and 13 might be orthologous to PA55410 in P. aeruginosa PA14 [7], the strain known to be highly virulent, and PA0688 from P. aeruginosa PAO1.

We next amplified and sequenced genes analogous to *pa5369* in MDR strains 1, 13, and 25 (GenBank Accession numbers EF601157, EF601158, and EF601159). We determined them to be very conserved with few differences in nucleotide sequences that did not affect amino acid sequences which were 100% identical to PA5369 in P. aeruginosa PAO1 (http://www.pseudomonas.com/). We therefore created anti-PA5369 antibodies against the specific peptide 192–212 KEEALCKGDFRPNVNEQPGS that was chosen based on hydrophobicity, surface probability, flexibility, and antigenic index, as well as the Advanced BLAST Search for the absence of significant homology to other P. aeruginosa proteins. Antibodies were subjected to affinitive purification using the native peptide 192–212, and then used to detect appendage-like structures in the clinical isolates. We first performed immunobloting of cell surface structures using anti-192–212 peptide antibodies, now referred to as anti-PA5369 antibodies, and found high antibody affinity to cell surface proteins of strain MDR25, moderate affinity to strains MDR1 and MPAO1, and minimal affinity to strain MDR13 (Figure 2C). These data corresponded to the results of the SDS-PAGE demonstrating an abundance of the 32 kDa protein band in strain MDR25 but not in strain MDR13. The specificity of the anti-PA5369 antibody was confirmed by examining both sheared appendages and bacterial pellets in wild-type MPAO1, PA5369 mutant, and clinical strain MDR25 (Figure 2D). Results demonstrated that anti-PA5369 recognized abundant amounts of protein in both the cell pellet and sheared appendages in strain 25. In strain MPAO1, antibodies recognized an abundant amount of protein in the cell pellet but a low amount in sheared appendages. No recognizable protein in either the cell pellet or sheared surface fractions in the MPAO1 mutant ΔPA5369 was observed. ELISA assays performed with sheared proteins from different clinical isolates (Figure 2E) demonstrated the presence of highly abundant PA5369-like protein in MDR clinical strains 25, 27, and 28, all of which share the same genotype 20. Interestingly, anti-PA5369 antibodies recognized significantly lower amount of proteins in sheared fractions isolated from other clinical isolates previously shown to be unable to alter the epithelial resistance of Caco-2 monolayers (see Table 1) [2]. Another interesting finding was the presence of PA5369 in sheared fractions of MPAO1 by both ELISA (Figure 2E) and immunobloting (Figure 2D), although much lower in abundance compared to the highly adherent strains MDR25 and MDR1. PA5369 has been predicted by COG (Clusters of Orthologous Groups, http://www.pseudomonas.com/) to be PstS, a phosphate transport system substrate-binding protein whose expression in P. aeruginosa is induced at phosphate concentrations < 1 mM [8–12]. In order to determine if the formation of appendages in clinical isolate 25 was phosphate dependent, we suspended a single colony in 10% glycerol, and plated equal amounts on either PIA that we measured to contain 300 μM of phosphate or PIA supplemented with 1 mM K-phosphate buffer, pH 7.0. Cells grown on these plates were analyzed for the presence of appendages by transmission electron microscopy (TEM) and immunobloting. TEM images clearly demonstrated the absence of appendages in strain MDR25 grown on phosphate-enriched PIA (Figure 2F) and an abundance of appendages in the same strain grown on PIA only (Figure 2G). We also noted the differences in colony phenotype when smooth surface colonies appeared on high Pi media (Figure 2Fa), and rough surface colonies appeared on low Pi media (Figure 2Ga). Immunoblot analysis (Figure 2H and 2I) demonstrated the absence of proteins recognized by anti-PA5369 antibody on phosphate rich media versus their abundance on phosphate poor media in both sheared cell surface fractions (Figure 2H) and cell pellets (Figure 2I). Finally, we performed immuno-gold electron microscopy of strain MDR25 to confirm the presence of PstS protein on the appendages. Whole cells of strain MDR25 were directly harvested from PIA plates and incubated with anti-PA5369 antibody followed by incubation with gold-labeled goat anti-rabbit antibody. Figure 2J demonstrates gold spots localized on the cell surface structures in strain MDR25. Gold spot localization was not observed in negative controls performed in the absence of primary anti-PA5369 antibodies (data not shown). We noted the fragility of these appendages on EM as detached and fragmented appendages were observed (see Figure S3).

### The PstS-Like Protein Contributes to the Ability of P. aeruginosa MDR25 and MPAO1 to Adhere to and Disrupt the Integrity of Cultured Intestinal Epithelial Cells

**Figure 3:**
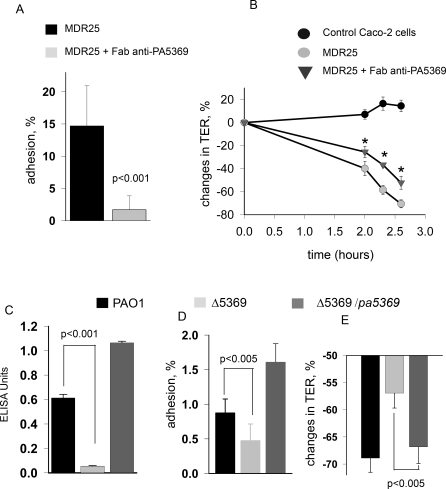
PA5369 Is Involved in Adhesiveness and Ability of MDR25 and MPAO1 to Disrupt Epithelial Integrity (A) Anti-PA5369 antibodies decrease the adhesiveness of strain MDR25 to Caco-2 monolayers*.* Strain MDR25 was grown on PIA plates for 2 days, bacteria were harvested, resuspended in PBS to a cell density of 5.0, and 2 μl was added to the apical surface of confluent Caco-2 cells. The Fab fragments of anti-PA5369 antibody at dilution 1:100 were included in the DMEM media (when needed) at the point of inoculation. Following 1 hour of incubation at 37°C, 5% CO_2_, the percentage of adherent cell was calculated. Data are mean ± SD (n = 3), *p* < 0.001 (Student t-test). (B) Anti-PA5369 antibodies attenuate the ability of strain MDR25 to disrupt Caco-2 monolayer integrity. Strain MDR25 was grown on PIA plates for 2 days, bacteria were harvested, resuspended in PBS to a cell density of 5.0, and 2 μl was added to the apical surface of confluent Caco-2 cells grown in transwells. The Fab fragments of anti-PA5369 antibody at dilution 1:100 were included in the DMEM media at the point of inoculation. Transepithelial resistance (TER) was measured dynamically as previously described [2]. The control group reflects the TER of Caco-2 cells in the absence of MDR25. Data are mean ± SD (*n* = 3), *p* < 0.005 (Student t-test). (C) Detection of surface associated PstS by ELISA in strain MPAO1 and derivative strains. Data are mean ± SD (*n* = 3), *p* < 0.001 (Student t-test). (D) PA5369 is involved in the adhesiveness of MPAO1 to Caco-2 cell monolayers*.* MPAO1 and its derivative strains were grown in TSB overnight, and 2 μl added to the apical surface of confluent Caco-2 cells. Following 1 hour of incubation at 37°C, 5% CO_2_, the percentage of adherent bacteria was calculated as previously described [2]. Data are mean ± SD (*n* = 3), *p* < 0.005 (Student t-test). (E) PA5369 is involved in the ability of MPAO1 to disrupt Caco-2 monolayer integrity. MPAO1 and its derivative strains were grown in TSB overnight, and 2 μl added to the apical surface of confluent Caco-2 cells grown in transwells. Transepithelial resistance (TER) of Caco-2 cells was measured after 7 hours of co-incubation with P. aeruginosa strains. Data are mean ± SD (*n* = 3), *p* < 0.005 (Student t-test)

In order to determine the contributory role of the PA5369-like protein on the ability of MDR P. aeruginosa to adhere to and disrupt barrier function of cultured intestinal epithelial cells, we examined the effect of anti-PA5369 antibodies on the adhesiveness and barrier disrupting capability. In order to avoid the non-specific interference of whole antibodies, we purified Fab fragments of anti-PA5369 antibodies to use in these experiments. Using stain MDR25, we performed adhesion assays to Caco-2 monolayers and determined the transepithelial resistance (TER) of Caco-2 cells, a measure of barrier function, in the presence or absence of Fab fragments of anti-PA5369 antibodies. Both the adhesiveness of strain MDR25 to Caco-2 monolayers (Figure 3A) and the ability of strain MDR25 to disrupt the TER of Caco-2 monolayers (Figure 3B) were significantly attenuated when pre-incubated with the Fab fragments of anti-PA5369 antibodies.

In order to determine if PstS could influence the ability of non-multi-drug resistant strains to alter intestinal barrier function, we performed complementary experiments using P. aeruginosa MPAO1 and its derivative mutant ΔPA5369 [13]. The mutant ΔPA5369 was complemented with the *pa5369* gene on a multi-copy plasmid pUCP24 (Δ5369/*pa5369*). First strains were verified for the presence of surface-associated PstS by ELISA using specific anti-PA5369 antibodies (Figure 3C). In order to determine if PstS contributed to the adhesiveness of MPAO1, we apically inoculated Caco-2 monolayers with P. aeruginosa strains and assessed the degree of adhesiveness after one hour of co-incubation. We observed the adhesiveness of MPAO1 to cultured intestinal epithelial cells to be as low as 1% of the initial inoculum; an effect that was further decreased with the mutant strain Δ5369 (Figure 3D). Strain Δ5369/*pa5369* demonstrated increased adhesiveness to Caco-2 cells compared to both the wild type and 5369 mutant (Figure 3D). Reiterative experiments were then performed to assess the ability of the strains to alter epithelial barrier function, as measured by TER of Caco-2 cells. We have previously reported strain MPAO1 to display low virulence against Caco-2 monolayers (∼5% decrease in TER at 3 hours) compared to clinical strain MDR25 (∼70% decrease in TER at 3 hours) (see Table 1). However at later time points (7 hours) strain MPAO1 decreased resistance of Caco-2 monolayers by 60%–70%. Therefore, we measured TER after 7 hours of co-incubation of Caco-2 cells using MPAO1 and its derivatives and found that strain Δ5369 was significantly attenuated in its ability to decrease the TER of Caco-2 monolayers (Figure 3E). Complementation of Δ5369 with *pa5369* gene restored its effect to decrease the TER of Caco-2 cells similar to the wild type PAO1 (Figure 3E).

### Smooth Versus Rough Appearing Colonies of MDR25 Differentially Express Surface-Exposed PstS and Differentially Induce Mortality in a Mouse Model of Gut-Derived Sepsis

**Figure 4:**
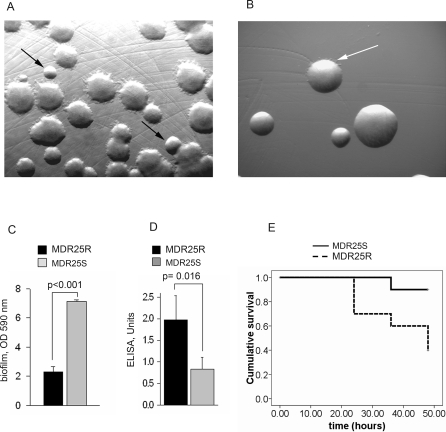
Colony Morphology Variation in MDR25 Correlates with Production of Outer Surface-Exposed PstS and Virulence (A) The appearance of smooth edged colonies as shown by black arrows in MDR25 grown on PIA, Gm 50 μg/ml. (B) A single smooth colony was isolated, cultivated in PB, Gm 50 μg/ml, and plated on PIA, Gm 50 μg/ml. The appearance of rough edged colony is shown by the white arrow. (C) Biofilm production by rough (MDR25R) and smooth (MDR25S) strains grown in PB, Gm 50 μg/ml. (D) Expression of outer surface PstS in MDR25R and MDR25S grown on PIA, Gm 50 μg/ml. (E) Kaplan-Meier survival curves of mice intestinally inoculated with MDR25S or MDR25R demonstrating attenuated killing effect of MDR25S. *n* = 10/group, *p* = 0.021.

We incidentally noticed the spontaneous appearance of smooth colonies among rough-edged colonies of MDR25 when grown on PIA where the phosphate level (Pi) was determined to be ∼300 μM (Figure 4A, black arrows). When smooth colonies were isolated and re-plated on PIA, the rough-edged colonies re-appeared interspersed among smooth colonies (Figure 4B, shown by white arrow) suggesting the possibility of colony phase variation. We also noted that rough (MDR25R) and smooth (MDR25S) colonies were distinct in their production of biofilm, whereby MDR25S produced significantly greater amounts of biofilm compared to MDR25R (Figure 4C). Growth curves for MDR25S and MDR25R were similar in liquid *Pseudomonas* broth (see Figure S4). We next examined smooth and rough colonies for their PstS content on surface sheared fractions by ELISA using anti-PA5369 antibodies and determined that smooth edge colonies produced significantly less PstS compared to rough colonies (Figure 4D). Finally, we determined if MDR25R and MDR25S differentially induced mortality in mice using an established model of lethal gut-derived sepsis [14,15]. This model involves creating a surgical stress with a 30% hepatectomy and simultaneous intestinal exposure to P. aeruginosa via direct injection into the cecum [15]. This model is of particular clinical relevance as it is well established that surgical hepatectomy results in severe hypophosphatemia [16]. Rough and smooth colonies were suspended in 10% glycerol at OD 0.25 (600 nm) and injected into the cecum at the time of hepatectomy. Mice were followed for 48 hours for mortality. Results demonstrated that mice injected with the smooth edged, PstS poor strain MDR25S displayed 10% mortality at 48 hours whereas mice injected with the rough edged PstS rich strain MDR25R, displayed 60% mortality at 48 hours (Figure 4E). Data were analyzed using Kaplan-Meier survival curves with SPSS software, *n* = 10/group, *p* = 0.021.

### Phosphate Supplementation Protects Mice from Lethality Following Intestinal Exposure to either MDR25R or MDR1

**Figure 5:**
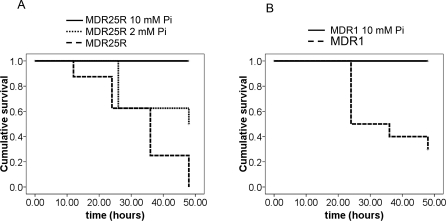
Intestinal Phosphate Supplementation Prevents the Lethality of MDR Strains in Mice Seven-week-old male C57BL6 mice were given either water (no phosphate supplementation), or 2 mM PBS solution (2 mM phosphate supplementation), or 10 mM PBS solution (10 mM phosphate supplementation) for 36 hours prior to hepatectomy. P. aeruginosa MDR25 was grown overnight in *Pseudomonas* broth (PB) containing Gm, 50 μg/ml, cells were collected by centrifugation, resuspended in either water containing 10% glycerol (group 1, *n* = 8), or 2 mM PBS (group 2, *n* = 8) or 10 mM PBS (group 3, *n* = 5) to final OD_600 nm_ 0.2, and 200 μl of bacterial solutions were injected into cecum immediately after hepatectomy. The abdomen was closed and mice were allowed to drink either water (group 1) or 2 mM PBS (group 2) or 10 mM PBS (group 3) and followed for mortality. Data were analyzed using Kaplan-Maier survival curves with SPSS software and significance tested by Log-rank (Mantel-Cox) *p* = 0.004. Reiterative experiments were performed with the clinical isolate MDR1 with the exception that only 10 mM of phosphate supplementation was tested (*n* = 10/group, *p* = 0.001).

We determined if phosphate supplementation in mice subjected to a 30% hepatectomy, could prevent lethality due to MDR25R or MDR1 by performing reiterative experiments in which mice were fed varying concentrations of phosphate ([Pi]). Group 1 (*n* = 8) were fed water only, Group 2 (*n* = 8) were fed 0.2x PBS (phosphate buffered saline, pH 7.4, [Pi] = 2 mM) as their drinking water, and Group 3 (*n* = 5) were fed 1x PBS ([Pi] =10 mM) as their drinking water for 36 hours before surgical hepatectomy and injection of bacteria. In addition, prior to injection of MDR25R into the cecum, bacteria were suspended in either water containing 10% glycerol (group 1) or 0.2x PBS (group 2) or 1x PBS (group 3). Results shown in Figure 5A demonstrate that MDR25R caused 100% mortality within 48 hours when mice drank water only (Group1) whereas mice drinking a water solution containing 2 mM phosphate (Group 2) had significantly decreased mortality (50%) while mice drinking a water solution containing 10 mM phosphate (Group 3) had no mortality (100% survival). Data were analyzed using Kaplan-Meier surviving curves in SPSS software, *p* = 0.004. Similar results were found in reiterative experiments with the clinical isolate MDR1 (Figure 5B) (*n* = 10, *p* = 0.001).

### The Alternative Type II Secretion System *hxc* Contributes to Outer Surface Expression of PA5369

**Figure 6:**
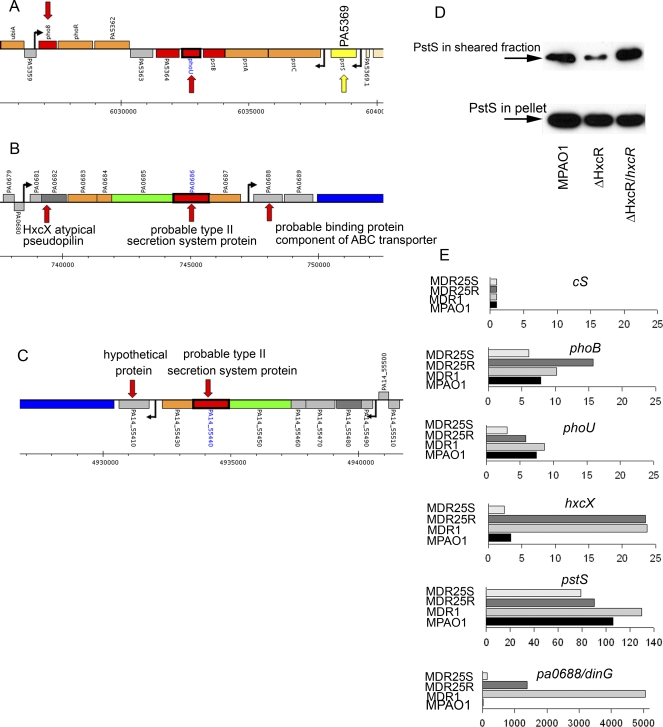
Alternative Type II Secretion System Is Involved In Outer Surface Expression of PstS (A) Clustering of *pstS pa5369* to the P. aeruginosa PAO1 genome. (B) Clustering of *pstS pa0688* to the P. aeruginosa PAO1 genome. (C) Clustering of *pstS pa14–55410* to P. aeruginosa PA14 genome. (D) Immunoblot analysis of pellet and sheared surface proteins from strains MPAO1, MPAO1 derivative PA0686 (ΔHxcR) mutant, and ΔHxcR complemented mutant. Strains were grown on PIA, and colonies collected and suspended in 200 μl PBS containing protease inhibitor cocktail (Sigma) up to final density of 5.0 (600 nm) followed by vortexing for 2 min and centrifuging at 5,000*g*, 5 min. Sheared material in 10 μl of supernatant was denatured by boiling with 10 μl sample buffer and 10 μl was loaded on gel wells to be separated by 10% glycine SDS-PAGE. The pellet was resuspended in 50 μl of PBS containing protease inhibitor cocktail, and 150 μl of BugBuster Master Mix (Novagen) was added to lyze the bacterial cells. Debris was removed by centrifugation, and 10 μl of supernatant was denatured by boiling with 10 μl sample buffer and 10 μl was loaded on gel. PstS protein was identified by immunoblot using anti-PA5369 antibody. (E) Expression of *pstS* and *pstS*-related genes under phosphate limitation determined by Real-Time RT-PCR. Gene expression was performed in triplicate with data representing the mean, and three independent experiments run showing similar expression patterns. The (-RT) controls were determined to be at the level of blank control (no template included).

The outer surface expression of PstS PA5369 observed in the current study is at variance with its previously reported characterization as a periplasmic protein. In order to clarify this we hypothesized that knockout of adjacent low phosphate responsive elements might impair the surface expression of PstS. PstS PA5369 is clustered to the phosphate ABC transporter locus (Figure 6A). Based on the KEGG SSDB (Kyoto Encyclopedia of Genes and Genomes Sequence Similarity DataBase http://www.genome.jp/kegg/ssdb/) search, PstS PA5369 can be considered as a paralogous protein of PstS PA0688 in P. aeruginosa PAO1 that is clustered to the alternative type II secretion locus (Figure 6B). According to recent data [17], PA0688 is characterized as alkaline phosphatase that is secreted by the alternative type II secretion system Hxc induced under low phosphate conditions. We therefore hypothesized that *hxc* might also play a role in the outer surface expression of PstS PA5369 and performed immunoblot analysis of sheared appendages in strains P. aeruginosa PAO1 and its derivative mutant ΔHxcR (PA0686). As shown in Figure 6D, the ΔHxcR mutant was attenuated in the production of the outer surface but not intracellular PA5369 suggesting at least partial involvement of *hxc* system to present PstS on outer surface appendages. Moreover, complementation of the mutant with *hxcR* restored its ability to express outer surface PstS (Figure 6D), confirming involvement of the Hxc system. N-terminal sequence of proteins from sheared fractions of the MDR isolates 1 and 13 by Blast Search correspond to PA14–55410 which is orthologous to PA0688 and clusters to the *hxc* system (Figure 6C). We amplified and sequenced the corresponding gene in strain MDR1, and found that the protein encoded by this gene had 90% identity to PA14 55410 from P. aeruginosa PA14, 45.3% identity to PA0688 from P. aeruginosa PAO1, and 64.3% identity to the human plasma phosphate-binding protein HPBP which is classified as a DING protein [18–20]. We therefore named the protein from MDR1 as DING and its respective gene *dinG* (GenBank Accession number EF616488). We next performed experiments to determine the expression level of *pstS* and related genes from the phosphate ABC transporter system, *pstS pa5369*, *phoB* and *phoU*, as well as *pstS* and related genes from *hxc* system, *pa0688*, *dinG*, and *hxcX*, a gene of *hxc* operon (Figure 6A–6C). We examined four strains: MPAO1, MDR1, MDR25R, and MDR25S. Strains were grown overnight on PIA and PIA complemented with 10 mM K-Ph buffer, pH 7.0, and RNA was directly isolated from cell colonies. Results are presented in Figure 6E. While the expression of the housekeeping enzyme citrate synthase demonstrated no significant change in response to phosphate limitation, the expression of all genes tested was increased in response to low phosphate media with each expressing a distinct pattern. While a similar increase in *phoU* expression was observed between all strains, only a modest increase in *phoB* expression was detected in MDR25R compared to other strains. Similarly only a modest increase in *pstS pa5369* expression was observed in the MDR isolates compared to MPAO1. The most intriguing finding however was that, although *pstS pa5369* expression was similar between the rough (high outer surface PstS) and smooth (low outer surface PstS) colony variants, a dramatic difference in expression was observed in the *hxc* operon. In fact, *hxcX* expression was ten times higher in MDR25R compared to MDR25S. The low response of *hxcX* to phosphate limitation was also observed in MPAO1 strain compared to MDR25R and MDR1. Although the expression of *pa0688*, the orthologous of DING protein in MPAO1, was 20-fold higher at low phosphate concentrations, this effect was small compared to that observed for MDR strains where a 150-fold increase was observed with MDR25S, a 1,400-fold in MDR25R, and 5,000-fold increase in expression in MDR1.

## Discussion

Numerous reports have documented that the rise in MDR nosocomial pathogens continues to threaten hospitalized patients despite the implementation of various countermeasures including isolation techniques and antibiotic de-escalation measures [21]. While the mere culture of a MDR resistant pathogen such as P. aeruginosa is perceived to be a real and present danger to patients primarily because it cannot be readily eliminated by antibiotics, the evidence that antibiotic resistance itself confers a more virulent phenotype is highly variable. Our previous work on screening consecutively isolated MDR strains of P. aeruginosa from critically ill hospitalized patients demonstrated that strains express extremely polar virulence phenotypes against the intestinal epithelium from those that are essentially inert, to those that are highly motile, adhesive, and destructive [2]. In fact among the consecutively collected strains in this series, only a minority of strains displayed a virulent phenotype against the intestinal epithelium (∼15%). A better understanding of the virulence determinants of MDR P. aeruginosa and their mechanism of action against the intestinal epithelium is important given the high prevalence of colonization of this organism in the intestinal tract of critically ill and immuno-compromised patients [22–24].

Human critical illness represents a unique ecological niche for P. aeruginosa because of its prolonged exposure to antibiotics and physiologic disturbances that have no historical precedent in terms of host survival. Extensive life sustaining measures employed during the care of the critically ill such as the use of gastric acid suppression therapy, vasoactive agents that result in profound luminal hypoxia, continued use of opioids that impair the ability of the intestinal tract to excrete non-commensal pathogens, and the delivery of highly processed artificial nutrition, all favor the exposure of pathogens like P. aeruginosa to a composite of environmental cues that can directly activate its virulence circuitry [25–29]. In this regard a major environmental cue within the intestinal tract that could shift the virulence of P. aeruginosa to that of a more virulent phenotype against the epithelium may be low extracellular phosphate which is often present during severe critical illness [16,30–32]. Hypophosphatemia is reported to be present following a variety of physiologic stress states such as myocardial infarction [33], ischemia-reperfusion injury [33], major liver resection [16], use of insulin to control hyperglycemia [34,35], use of intravenous nutrition [36,37], and during sepsis [38]. In such circumstances, phosphate depletion appears to be severe and an independent predictor of mortality due to infection and sepsis [38]. While the mechanisms for this observation are unknown, it is possible that colonizing strains of P. aeruginosa present in the intestinal lumen of critically ill patients become activated to express a more virulent phenotype against the intestinal epithelium in response to low phosphate concentrations resulting from surgical injury and catabolic stress.

In the present study, we determined that MDR P. aeruginosa clinical strains displaying a high degree of virulence against cultured intestinal epithelial cells express an extraordinary amount of surface-associated PstS proteins orthologous to PA5369 from P. aeruginosa PAO1 and PA14 55410 protein from P. aeruginosa PA14. The observation that PstS on appendages contributes to intestinal epithelial adherence coupled with its known role as a phosphate binding protein, raises the possibility that the PstS present on appendages might facilitate the ability of MDR P. aeruginosa to acquire phosphate from intracellular stores within the host. This latter effect appeared to be dependent on the presence and expression of the alternative type II secretion system Hxc, which itself is activated in the presence of low phosphate [17]. This finding establishes a link between the phosphate binding ABC transporter and the Hxc system in P. aeruginosa. Simultaneous expression of both systems is necessary for the formation of outer surface PstS-rich appendages as neither ΔPstS nor ΔHxcR mutants produce them in P. aeruginosa MPAO1. Both the PstS and the Hxc systems are highly inducible in MDR clinical isolates that express a particularly adhesive and barrier disrupting phenotype against intestinal epithelial cells. In this regard, certain MDR *P. aeruginosa s*trains may have adapted unique genetic changes in response to unusually harsh selective pressures that typify critically ill humans including multiple antibiotic use, severe hypoxia, and the ability to sustain life with prolonged intravenous nutrition. Among such changes may be the ability to acquire phosphate and other nutrients from within host cells given that physiologic stress and tissue injury are know to shift phosphate into the intracellular compartment resulting in severe hypophosphatemia [39]. Under such circumstances outer surface expression of PstS on appendages may confer an evolutionary advantage to P. aeruginosa by expressing phosphate acquiring structures capable of scavenging intracellular phosphate at arm's length from the host immune system.

Data from the present study are not the first to demonstrate that PstS is secreted by bacteria during nutrient manipulation of the media. For example, it has been recently reported that Streptomyces lividans secretes PstS into liquid cultures containing high concentrations (>3 %) of certain sugars, such as fructose, galactose, and mannose [40]. Another example in which PstS has been shown to be secreted is PA0688 protein in P. aeruginosa PAO1. Inquiry of the PA0688 protein into the “Clusters of Orthologous Groups (COG) Program” predicted it to be PstS. However PA0688 clusters to the *hxc* loci and has been shown to be secreted by the Hxc system under phosphate depleted conditions and functions as alkaline phosphatase [17]. In the current study, the proteins identified in MDR P. aeruginosa orthologous to PA0688 were expressed several hundred fold higher than in MPAO1. That sequence analysis revealed this protein in MDR1 to belong to DING proteins is intriguing, given that the origin and function of DING proteins have remained a focus of speculation. DING proteins are characterized by their N-terminal DINGGGATL-sequence and are highly conserved in both animal and plants, although they are more variable as microbial proteins [19,41–43]. There are some functional similarities between DING proteins from pro- and eukaryotes including structural homology with phosphate-binding proteins [19,43]. It has been hypothesized that pathogenic or symbiotic bacteria might acquire the DING gene via horizontal gene transfer from eukaryotes in order to sense and respond to host signals or to modify intercellular signaling pathways in host cells [42]. Others have suggested that DING proteins do not exist in eukaryotes at all, and that their detection in human tissues has been a result of microbial contamination or infection [41]. Based on the codon usage analysis of DNA, it has been assumed that DING sequences found in eukaryotes are of *Pseudomonas* origin [41]. Further work is in progress to characterize the role of the DING-protein related appendages found in this series of MDR clinical isolates, the results of which may add to our understanding of the impact of bacterial DING proteins on the modulation of signal transduction in animals.

The gene *pstS pa5369* is part of the *pst* operon encoding a specific phosphate transport system that is activated under low phosphate conditions. The high affinity phosphate transport system *pst* belongs to the Pho regulon that is controlled by the two-component regulatory system PhoB/PhoR, which responds to local phosphate concentration. PhoB/PhoR is highly conserved and widely present in Gram negative and Gram positive microorganisms. In addition, PhoB/PhoR controls the expression of multiple genes [44,45] many of which are involved in phosphate uptake and metabolism and various other metabolic pathways such as the de novo biosynthesis of NAD [46], the initiation of chromosome replication [47], the acid shock response [48], the RpoS-mediated stress response [49], and AMP hydrolysis [44,50]. The phosphate regulon might be also involved in the activation of quorum sensing in P. aeruginosa as evidenced by the recent observation that the transcriptional activation of *rhlR* and production of PQS and pyocyanin develop during phosphate limitation [12]. Furthermore, a link between the expression of the ABC phosphate transporter and penicillin resistance in Streptococcus pneumoniae has been reported thereby proposing a novel role for PstS [51]. These investigators reported that the *pstS* gene product was overproduced in resistant isolates, the inactivation of which resulted in penicillin sensitivity [51]. Further evidence linking PstS to antibiotic resistance has been demonstrated in fluoroquinolone resistant Mycobacterium smegmatis [52] where amplification of the phosphate specific transporter suggested that the efflux mediated fluoroquinolone resistance might be an intrinsic function of the Pst system [52–54]. Thus is it plausible that the development of multi-drug resistance in P. aeruginosa clinical isolates MDR1, MDR13, and MDR25 might be related to the overproduction of PstS proteins.

Mouse lethality experiments from the present study strongly suggest a significant role for PstS in the virulence of MDR25R P. aeruginosa virulence *in vivo*. The importance of PstS in *in vivo* virulence has been previously addressed in various models including a mouse infection model using Mycobacterium tuberculosis and *pstS1* and *pstS2* knockout strains [55], a fish infection model using Edwardsiella tarda, a facultative aerobic enterobacterium that causes hemorrhagic septicemia in fish and gastrointestinal infections in humans [56], and a chicken infection model using Escherichia coli O78, an organism associated with extraintestinal infections and septicemia in poultry, livestock, and humans [57].

In summary, we have identified PstS-rich appendage-like structures on the outer surfaces of selected strains of MDR P. aeruginosa that confer a highly adhesive and virulent phenotype against cultured intestinal epithelial cells. Further characterization of these appendages and better understanding of their molecular regulation are needed to fully define their role in the virulence of multi-drug resistant P. aeruginosa. The observation that critical virulence factors such as PstS in P. aeruginosa are highly responsive to environmental phosphate, in conjunction with the observation that intestinal phosphate repletion completely prevented mortality in surgically injured mice exposed to MDR strains, underscores the importance of recognizing intestinal phosphate depletion following catabolic stress and a possible strategy of intestinal phosphate loading as a countermeasure against colonizing strains of P. aeruginosa that are resistant to all conventional antibiotics.

## Materials and Methods

### Bacterial isolates.

The consecutively collected clinical strains of MDR P. aeruginosa used in the present study (Table 1) have been characterized and described previously [2]. P. aeruginosa strains MPAO1, MPAO1 mutant ΔPA5369 (PA5369:: ISphoA/hah, ID 29772, and MPAO1 mutant ΔPA0686 (PA0686:: ISphoA/hah, ID 957) were obtained from the P. aeruginosa mutant library [13]. The mutant ΔPA5369 was complemented with *pa5369* gene to create strain ΔPA5369/ *pa5369*, and the mutant ΔPA0686 was complemented with DNA comprising *pa0686* plus *pa0687* genes to create the strain ΔPA0686/ *pa0686*-*pa0687*. The MDR clinical isolates were routinely subcultured from frozen stocks on *Pseudomonas* isolation agar (PIA) containing Gm, 50 μg/ml. Note that strain 25, herein referred to as MDR25, and all strains of genotype 20 (see Table 1) did not grow on rich media (LB- liquid or agarized) or TSB (liquid or agarized), and did not grow in the specially designed phosphate limited liquid media described by Hancock [58]. We observed strain MDR25 growth was best supported in *Pseudomonas* broth that we determined to contain 2 mM Pi, and PIA determined to contain ∼0.3 mM Pi.

### Assessment of barrier function of cultured human intestinal epithelial cells.

Human intestinal epithelial cells Caco-2_bbe_ were grown to confluence in 0.3 cm^2^ transwells (Costar), and their barrier function was assessed by measuring the transepithelial electrical resistance (TER) to a fixed current across cells as previously described [2]. All experiments were performed in triplicate.

### Adhesiveness.

Adhesiveness of P. aeruginosa to Caco-2_ bbe_ cells was determined as previously described [2]. All experiments were performed in triplicate.

### Biofilm.

Biofilm formation was assayed as described with modifications [59]. Briefly, P. aeruginosa MDR clinical strains were grown overnight in 2 ml of PB, Gm 50 μg/ml in 15 ml culture tubes at 37°C, 200 rpm (C24 Incubator Shaker, New Brunswick Scientific, Edison, NJ). The wells were then rinsed thoroughly with water and the attached material was stained with 3 ml of 0.1% crystal violet, washed with water, and solubilized in 3 ml of ethanol. Solubilized fractions were collected and absorbance measured at 550 nm. All experiments were performed in triplicate.

### Identification of protein on novel surface structures.

P. aeruginosa strains were grown on PIA plate, than bacteria were harvested, suspended in PBS, and surface-associated structures were sheared by vigorous vortexing for 2 min. After centrifuging at 5,000*g*, for 5 min, proteins in the supernatant were separated using 10% Tris-glycine SDS-polyacrylamide gel and detected by Coomassie brilliant blue staining. For amino-terminal peptide sequence analysis, the proteins were electroblotted onto polyvinylidene fluoride (PVDF) membranes, and sequenced by Edman degradation chemistry using an Applied Biosystems Procise 492 HT Protein Sequencer (Applied Biosystems, Foster City, CA) at the Mayo Proteomics Research Center (Mayo Clinic College of Medicine, Rochester, MN).

### Antibodies and immunoblot analysis.

Polyclonal rabbit antiserum against 192–212 peptide KEEALCKGDFRPNVNEQPGS of PA5369 (anti-PA5369) was produced in rabbits (SynPep Corporation, Dublin, CA). Anti- PA5369 antibodies were affinity purified by AminoLink Plus Immobilization Kit (Pierce) using 192–212 peptide to create an affinity column. For immunoblot analysis, proteins were electrotransferred from SDS-polyacrylamide gels to PVDF membrane (Immobilon-P, Millipore) and primed with affinity pure anti-PA5369 antibodies at 1:1,000 dilution. Affinity pure F(ab)_2_ fragments of anti-rabbit IgG conjugated with horseradish peroxidase (Jackson Immunological Res Lab) at 1:5,000 dilution was used as secondary antibody. Detection was performed using SuperSignal West Dura Extended Duration Substrate (Pierce).

### Enzyme-linked immunosorbent assay (ELISA).

P. aeruginosa strains were grown on PIA plates for 2 days, bacteria were harvested, suspended in PBS containing protease inhibitor cocktail (Roche) to create a cell density of 5.0 (OD 600 nm) in a total volume of 500 μl, and centrifuged at 6,000 rpm, 5 min. The pellet was resuspended in 500 μl PBS containing protease inhibitor cocktail, and vigorously vortexed for 2 min. Cell surface associated proteins were separated by centrifuging for 5 min at 5,000 *g*. After centrifugation, the supernatants were diluted (1:5) with carbonate-bicarbonate buffer (Sigma), and 200 μl/well was used for coating Maxisorp Loose Immuno-modules (Nunc). Plates were incubated at 4°C, overnight, washed with PBS, and unbound sites were blocked with 3% bovine serum albumin in PBS for 30 min at room temperature. Rabbit polyclonal affinity purified anti-5369 antibody (1:1,000) followed by HRP-labeled affinity purified F(ab)_2_ fragments of anti-Rb IgG (Jackson Immunological Research Laboratories) (1:5,000), and o-phenylaminediamine (Sigma) were used to detect PA5369-like protein at 450 nm optical density.

### Purification of Fab fragment of anti-PA5369 antibodies.

Polyclonal affinity purified rabbit anti-PA5369 antibody against 192–212 peptide of PA5369 were used to isolate Fab fragment by ImmunoPure Fab Preparation Kit (Pierce) accordingly to manufacturer protocol.

### Electron and immuno-electron microscopy.

Transmission electron microscopic analysis was performed as previously described [60] with minor modifications. Briefly, bacteria were grown for 48 hours on PIA media with/without Gm, 20 μg/ml. A drop of water was deposited on the edge of colony, and a Formvar-coated copper grid was immediately floated on the drop for 30 s, then rinsed with TE buffer (10 mM Tris-HCl, 1 mM EDTA, pH 8.0) and stained with a 1% aqueous solution of uranyl acetate. Samples were examined under 300 KV with a FEI Tecnai F30 electron microscope.

For immuno-gold labeling, 200 mesh formvar-coated nickel grids were rinsed with TE buffer, rehydrated with PBS for 30 min and blocked with 1% BSA for 30 min followed by transferring to anti-PA5369 antibodies diluted as 1:100 in 1% BSA. Incubation was allowed at a humidified chamber for 3.5 hours, at room temperature, followed by extensive washing with PBS, blocking with 0.1% BSA for 25 min, and incubating in the humidified chamber for 1 hour with goat anti-rabbit IgG conjugated with 10 nm gold particles (TED PELLA) at 1:10 dilution in 0.1% BSA. Grids were washed with PBS, fixed with 1% glutaraldehyde in PBS for 10 min, washed with water, and stained briefly with uranyl acetate and lead citrate. Air dried grids were examined under 300KV with FEI Tecnai F30.

### Construction of pUCP24/5369.

The *pa5369* gene was amplified using PAO1 DNA and primers forward *5369F EcoRI* 5' CCGGAATTCGATGAAACTCAAGCGTTTG 3'and reverse *5369R XbaI* 5' GCTCTAGACAAGTCACTGGATTACAG 3' and cloned in *E.coli*-P. aeruginosa shuttle vector pUCP24 [61] using *EcoRI* and *XbaI* restriction sites to create pUCP24/*5369* where *pa5369* is expressed from *Plac* promoter. The plasmid pUCP24/*5369* was electroporated in ΔPA5369 to create strain Δ5369/*5369*.

### Construction of pUCP24/0686–0687.

The DNA containing *pa0686* and *pa0687* was amplified using PAO1 DNA and primers forward *PA0686-744301F-EcoRI* 5' CCGGAATTCGCGCGGTACCGTTGG 3' and *PA0687-746961R-XbaI* 5' GCTCTAGACGGACTACTGGACCAGTTG 3'and cloned in *E.coli*-P. aeruginosa shuttle vector pUCP24 [61] using *EcoRI* and *XbaI* restriction sites to create pUCP24/0686–0687 under regulation of *Plac* promoter. The plasmid pUCP24/0686–0687 was electroporated in the MPAO1 mutant strain ΔHxcR (ΔPA0686) to create strain ΔHxcR/*0686–0687*.

### Amplification and sequencing of genes analogous to *pstS* in MDR clinical isolates of P. aeruginosa.

The forward primer *5369F EcoRI* 5' CCGGAATTCGATGAAACTCAAGCGTTTG 3'and reverse primer *5369R XbaI* 5' GCTCTAGACAAGTCACTGGATTACAG 3' were designed based on the sequence of P. aeruginosa PAO1 genome and used to amplify genes using genome DNA isolated from strains MDR 1, MDR 13, and MDR 25. The gene analogous to *pa14–55410* was amplified using genome DNA of strain MDR1, and primers *55410F EcoRI* 5'CCGGAATTCGATGTACAAGCGCTCTCTGAT 3' and *55410R XbaI* 5' GCTCTAGACAAG TTAGAGCGGACGGCCGAT 3' designed based on the sequence of P. aeruginosa PA14 genome. Amplified DNAs were cloned directly into pCR2.1 (Invitrogen), and the sequence was obtained using standard M13 Forward and M13 Reverse primers on an Applied Biosystems 3730XL genetic analyzer (University of Chicago, Cancer Research Center, DNA Sequencing & Genotyping Facility).

### Real-time PCR.

Strains MPAO1, MDR25R, MDR25S, and MDR1 were grown overnight on PIA and PIA supplemented with 10 mM K-Ph buffer, pH 7.0, and collected directly in the RNA protect buffer (Qiagen), and RNA isolation, DNA degradation, and cDNA synthesis were performed as previously described [27]. Real time PCR was performed on the ABI 7900HT System using SYBR Green qPCR SuperMix-UDG (Invitrogen), cDNA, and respective primers: for citrate synthase PA1580 gene *gltA*, PA1580–434 5' TCTACCACGACTCCCTGGAC 3' and PA1580–590 5' TTTTCCGCGTAGTTCAGGTC 3'; for PstS PA5369 gene *pstS*, PA5369–148 5' ACTCTGGCCAACCTGATGAC 3' and PA5369–335 5'CCGTACTTCTGCTCGAAAGC 3'; for phosphate uptake regulator PhoU PA5365 gene *phoU*, PA5365–523 5' CGCGAACTGGTCACCTACAT 3' and PA5365–711 5' CTCGACCTCTTCCTTCATGC 3'; for low phosphate response regulator PhoB PA5360 gene *phoB*, PA5360–7 5' GGCAAGACAATCCTCATCGT 3' and PA5360–164 5'CAGTCGAGCAGGATCAGGTC 3'; for PstS PA0688 gene *pa0688*, PA0688–693 5'GGTGAACATCAACAGCAACG 3' and PA0688–872 5'TAACCGACGATGGAGTAGCC 3'; for PstS analogous to PA14–55410) gene *dinG*, S1-DING-427 5' CTCTGCCGTTCAACAAGTCA 3' and S1-DING-604 5'CGGGTGAACAGTTCGGTAGT 3', for HxcX atypical pseudopilin PA0682 gene *hxcX* , PA0682–299 5' AAGACGAGCAGGGCAAGTT 3'and PA0682–454 5'GTGCATAGGAGGCGAGTACC 3'. 0.5 μg of RNA after DNAse treatment was converted to cDNA in 20 μl of reaction mixture (High Capacity cDNA Reverse Transcription kit, Applied Biosystems). The cDNA and RNA (-RT control) were diluted either as 1:50 (for *cS*, *pstS 5369*, *phoU*, *phoB*) or 1:10 (for *hxcX*) or 1:500 (for *pa0688* and *dinG*), and 5 μl of diluted mixture was used as a template added to 7.5 μl of master mix containing as manufactured (Invitrogen) SYBR green, ROX, and respective primers. The amplification was run in 384 well plates. Expression levels were calculated based on differences in Ct levels.

All primers were confirmed for DNA amplification using genome DNAs from MPAO1 and MDR clinical isolates prior the Real Time experiments.

### Mouse model of lethal gut-derived sepsis.

All experiments were approved by the Animal Care and Use Committee at the University of Chicago (Protocol IACUC 71744). Six-seven week old male C57BL6 mice were ordered from Harlan Spraque Dawley Animal facility and allowed at least four days for housing acclimation prior to experiments. The mouse model of gut-derived sepsis was performed as previously described [15] with following modifications. Mice drank either water (no phosphate supplementation), or 0.2x PBS (2 mM Pi), or 1x PBS (10 mM Pi ) for 36 hours prior to lethality experiments. Animals were anesthetized (ketamine 100 mg/kg, xylazine 10 mg/kg) intraperitoneally and a 30% hepatectomy was performed on the left lobe of the liver, and the bacterial suspension of P. aeruginosa clinical isolates MDR25 or MDR1 were injected directly into the distal ileum and cecum with a fine high gauze needle. The abdomen was closed in two layers with suture, and mice were allowed to drink either water or PBS but were given no food for 48 hours. Animals were followed for mortality and sacrificed when they appeared septic and moribund.

### Mouse lethality studies using rough and smooth colony variants.

Eight-week-old male C57BL6 mice were given only water for 36 hours prior to hepatectomy. P. aeruginosa MDR25S and MDR25R were grown overnight in *Pseudomonas* broth (PB) containing Gm, 50 μg/ml. Overnight cultures were diluted as 1:500–1:250 in water containing 10% glycerol, and 20–50 μl were plated on PIA, Gm, 50 μg/ml. After 24–36 hours of growth, cells were collected directly from plates using an Olympus SZX16 stereo microscope to insure proper collection of rough and smooth colonies. The cells were diluted in water containing 10% glycerol to OD_600 nm_ 0.25, and 200 μl of bacterial suspension was injected in the cecum of mice immediately after hepatectomy. The abdomen was closed, and mice were allowed drinking water. Mice were followed for mortality and sacrificed when they appeared septic and moribund. Data were analyzed using Kaplan-Meier surviving curves and SPSS software employing the Long-rank (Mantel-Cox) test for significance.

### Statistical analysis.

Statistical analysis of the data was performed with Student t-test using Sigma plot software and Kaplan-Meier survival curves using SPSS software.

## Supporting Information

Figure S1 TEM Images of MDR and Non-MDR P. aeruginosa
Images of MDR clinical isolates from Table 1 not included in the main body of paper plus non-MDR control P. aeruginosa strains (2) are presented in Figure. Bacterial cells grown on *Pseudomonas* isolation agar were processed for electron microscopy as described in Materials and Methods.
(A) Highly virulent MDR clinical isolates: MDR27 and MDR28.
(B) Non-MDR control strains: MPAO1, PA27853, PA190 environmental isolate, and PA103.
Novel appendage-like structures and their detached fragments are indicated by black arrows, type IV pili are indicated by white arrows, and flagella are indicated by grey arrows. Note the hyperpiliation of PA103.
(9.8 MB TIF)

Figure S2 TEM Images of MDR P. aeruginosa
Low and non-virulent MDR clinical isolates: MDR2, MDR6, MDR7, MDR11, MDR15, MDR16, MDR17, MDR19, MDR31, MDR33.
(9.7 MB TIF)

Figure S3 TEM and Immuno-Electron Microscopy Images of MDR25 (A) TEM images displaying detached structures of appendages shown by arrows.
(B) Immuno-electron microscopy with gold-labeled secondary antibody identifying PstS indicated by black arrows. Flagella are indicated by grey arrows. Note lack of antibody affinity to flagella.
(8.1 MB TIF)

Figure S4 Growth Curves of MDR25S and MDR25R in *Pseudomonas* BrothFigure 1
(933 KB TIF)

### Accession Numbers

PstS PA5369 (Pseudomonas aeruginosa PAO1), NP_254056; PA0688, probable binding protein component of ABC transporter (Pseudomonas aeruginosa PAO1), NP_249379; PA14_55410, Hypothetical, unclassified, unknown (Pseudomonas aeruginosa UCBPP-PA14), complete genome NC_008463; PhoB, two-component response regulator (Pseudomonas aeruginosa PAO1), NP_254047; HxcX, atypical pseudopilin (Pseudomonas aeruginosa PAO1), NP_249373; PA0686, probable type II secretion system protein (Pseudomonas aeruginosa PAO1), complete genome NC_002516; PhoU, phosphate uptake regulatory protein (Pseudomonas aeruginosa PAO1), NP_254052; citrate synthase (Pseudomonas aeruginosa PAO1), NP_250271; Pseudomonas aeruginosa PAO1, complete genome, NC_002516; ExoU (Pseudomonas aeruginosa), AAC16023; human plasma phosphate-binding protein (HPBP), P85173.

In the present study: PstS (Pseudomonas aeruginosa MDR1), EF601157; PstS (Pseudomonas aeruginosa MDR13), EF601158; PstS (Pseudomonas aeruginosa MDR25), EF601159; DING (Pseudomonas aeruginosa MDR1), EF616488.
